# Dental amalgam: An update

**DOI:** 10.4103/0972-0707.73380

**Published:** 2010

**Authors:** Ramesh Bharti, Kulvinder Kaur Wadhwani, Aseem Prakash Tikku, Anil Chandra

**Affiliations:** Department of Conservative Dentistry and Endodontics, Faculty of Dental Sciences, CSM Medical University (Erstwhile King George’s Medical College), Lucknow, India

**Keywords:** Dental amalgam, direct restoration, esthetics and tooth colored material

## Abstract

Dental amalgam has served as an excellent and versatile restorative material for many years, despite periods of controversy. The authors review its history, summarize the evidence with regard to its performance and offer predictions for the future of this material. The PubMed database was used initially; the reference list for dental amalgam featured 8641 articles and 13 publications dealing with recent advances in dental amalgam. A forward search was undertaken on selected articles and using some author names. For the present, amalgam should remain the material of choice for economic direct restoration of posterior teeth. When esthetic concerns are paramount, tooth-colored materials, placed meticulously, can provide an acceptable alternative. All alternative restorative materials and procedures, however, have certain limitations.

## INTRODUCTION

Dental amalgam is one of the most versatile restorative materials used in dentistry. It constitutes approximately 75% of all restorative materials used by dentists. It has served as a dental restoration for more than 165 years. There is still no adequate economic alternative for dental amalgam. The combination of reliable long-term performance in load bearing situations and low cost is unmatched by other dental restorative material. It has a myriad of uses: rather low technique sensitivity, self-sealing property and its longevity.

Although there is evidence of a decrease in its use in the world, amalgam’s cost, durability and ease of manipulation have persuaded many dentists to continue to use it as their first choice for restoring posterior teeth. However, care must be taken in the diagnosis of the type of restoration to be placed. Where there is so much loss of tooth structure that support of the tooth must be given by the restoration, a gold inlay may be indicated, although there are some instances where even very extensive restorations of amalgam may be the choice. The PubMed database was used initially; the reference list for dental amalgam featured 8641 articles and 13 publications dealing with recent advances in dental amalgam. A forward search was undertaken on selected articles and using some author names.

## HISTORY

Dental amalgam apparently was first used by the Chinese. Su Kung (659 AD) mentioned the use of a mixture in the Material Medica.[1] In Europe, Johannes stokers, a municipal physician in Ulm, Germany, recommended amalgam as a filling material in 1528.[2] Later, Li Shihchen (1578) chronicled a dental mixture of 100 parts mercury with 45 parts silver and 900 parts tin.[1] The next major historical reference to silver–mercury amalgam was made in France. Traveau described a “silver paste” filling material in 1826. He produced amalgam by mixing the silver coins with mercury.[3] In 1833, the Crawcours brothers introduced to America their “Royal Mineral Succedaneum” which was actually shaved French silver coins and mercury. They filled the tooth cavity by removing the diseased tooth tissue and placing the amalgam on occlusal surface without knowing any relation to dental anatomy. Amalgam’s disrepute initiated by these brothers led to the “Amalgam War” and to the promulgation by the American society of dental surgeons in 1845. The use of amalgam was considered as a malpractice, and an expulsion from the society of those members who would use it was demanded.[1]

In 1877, the first organized movement on behalf of amalgam, the new departure creed and its leader J. Foster Flagg, managed to change this attitude toward dental amalgams. Flagg published the results of his laboratory tests and 5-year clinical observation of new alloys with 60% of silver and 40% of tin as major constituents in 1881 and thus predated by some 15 years the work of G.V. Black.[4,5]

The universal acceptance of amalgam as a restorative material resulted from investigations of Black in 1895, 1896, 1908. By combining the principles of cavity design, extension of the cavity into “immune” areas and the development of an alloy with the composition of 68.5% silver, 25.5% tin, 5% gold, 1% zinc, Black advanced amalgams into modern times. S.S. White manufactured the first commercial alloy rich in silver, True Dentalloy (1900), in which gold was replaced by copper.[6–8]

Extensive studies of the setting reaction of dental amalgams, performed by Gayler in 1937, further elucidated the mechanism of setting of amalgam and influence of amalgam components on expansion due to the gamma-1 phase (Ag–Hg) and contraction due to the formation of gamma-2 phase (Sn–Hg).[9] Greener in 1979 claimed that there is misinterpretation of Gayler’s work regarding the concentration of Cu, that the concentration of Cu above 5% produced increase expension. What Gayler did say was that if Cu was substituted for tin so that the concentration of tin dropped below 25% expension could occur; but if Cu was substituted for silver so that tin concentration was maintained at 27%, no excess expension occurred. This confusion surrounding the concentration of Cu has resulted in a 25-25–30 year delay in the development of amalgams resistant to corrosion.[10]

In 1959, Dr. Wilmer Eames recommended a 1:1 ratio of mercury to alloy, thus lowering the 8:5 ratio of mercury to alloy that others had been recommending.[11] In 1962, a spherical particle dental alloy was introduced.[12] This was followed in 1963 by a high copper dispersion alloy system that proved to be superior to its low copper predecessors.[13] Although this performance was theorized to be the result of dispersion strengthening of the alloy, researchers discovered that the additional copper combined with the tin, creating a copper–tin phase that was less susceptible to corrosion than the tin–mercury (gamma-2) phase found in low copper alloys.[14,15]

Introduction of new atomization process in the manufacture of dental amalgams led to a dramatic improvement in the quality and ease of manipulation with this material. This process involves spraying of the molten alloy into a chamber containing an inert gas by a patented atomization process.[16] The molten metal forms droplets which solidify. These spheres are then subjected to some heat treatment. Thus, spherical particles are formed.[16]

## DURABILITY OF AMALGAM RESTORATIONS

Recent research shows that amalgam restorations last longer than that was previously thought. The older generation of low-copper amalgams (before 1963) had a limited life span because they contained the gamma-2 phase that caused progressive weakening of the amalgam through corrosion.[17] Several clinical studies have demonstrated that high-copper amalgams can provide satisfactory performance for more than 12 years.[18–22] This appears to be true even for large restorations that replace cusps.[23] In addition, high-copper amalgams do not appear to require polishing after placement, as was recommended for low-copper amalgams, to increase their longevity.[24]

Plasmins *et al*.[25] evaluated the long-term survival of multisurface restorations and found that extensive amalgam restoration had no influence on the survival rate, which is in accordance with the results of a retrospective study by Robins and Summitt, who found 50% survival rate for 11.5 years.[26]

The satisfactory functioning of the extensive amalgam restorations over a long period of time results from the prevention of the most important traditional mechanical failure of amalgam restorations. These include marginal fracture, bulk fracture and tooth fracture.[27,28] The zinc and copper content of the alloy has been found to have a strong impact on the survival rates of amalgam restorations since it influences the corrosion resistance of the amalgam. High-copper amalgams have higher survival rates than conventional amalgams.[27]

Letzel[27] investigated survival and modes of failure of amalgam restorations retrospectively. The leading mode of failure was bulk fracture (4.6%), followed by tooth fracture (1.9%) and marginal ridge fracture (1.3%). For other reasons, 0.8% of the restorations failed.

## TOXICITY OF DENTAL AMALGAMS

The debate over the safety and efficacy of amalgam has raged since time immemorial. In recent times, it has reached such a feverish pitch that it seems to drown out all sounds of reason. Amalgam has served the dental profession for more than 165 years. Incidents of true allergy to mercury have been rare and attempts to link its usage with diseases like multiple sclerosis and Alzheimer’s disease have not been significantly proven, although there may be some association between amalgam restorations and oral lichenoid lesions.[29]

Marshall, in his review on dental amalgam, summed it up appropriately: “if some reported values of Hg release are extrapolated to clinical life times, the entire restoration could lose its Hg in short time. For example, a 500 mg amalgam restoration contains approximately 200–250 mg of Hg, and the entire quantity of Hg would be lost in 10,000 days if the Hg was released at the rate of 25 *μ*g/day. This estimate of release is of the order of magnitude reported in some studies of vapour release”.[30]

## COMPOSITION OF AMALGAM ALLOY

Composition of currently used alloy is silver 40–70%, tin 12–30% and copper 12–24%. It may also include indium 0–4%, palladium 0.5% and zinc up to 1%. Zinc prevents the oxidation of other metals in the alloy during manufacturing process.[19,21,31,32] Zinc also inhibits corrosion.[33] Some researchers believe that if zinc containing amalgam is contaminated with moisture, it causes delayed expansion.[34,35] Indium containing admixed high-copper amalgam exhibited a reduction in creep and increase in strength. Youdelis also found that less mercury is required for mixing amalgam when it contains indium in concentration up to 10%. The reason for lower mercury emission is that amalgam prepared with indium rapidly forms indium oxide and tin oxide films which reduce mercury release. Palladium reduces tarnish and corrosion.[13]

## DEVELOPMENTS IN CAVITY DESIGN

Traditionally, Black’s original preparation design called for extravagant extension with the intention of preventing recurrent caries. Overtime, improvements in knowledge have supported the more conservative cavity preparations. Some authors advocate extending the preparations into fissures, whether carious or not.[36–40] Smaller burs can be used to create preparations that involve the removal of only diseased and weakened enamel and dentin, and with the use of fissure sealants sound tooth can be preserved. A small diameter bur can be used to slightly open the fissures to be sealed to ensure access to sound enamel for etching and flow of a liquid resin to provide seal.[41]

Many studies have shown that smaller restorations last long.[42,43] Osborne and Gale evaluated 196 amalgam restorations 13–14 years after insertion. They found that cavity width was the single most significant factor for clinical survival. The wider restorations showed greater marginal fracture and a higher rate of replacement than narrow restorations. Other benefits associated with the success of smaller preparations include reduced occlusal stress on the margins and preservation of tooth strength.[44]

## RESIN COATED AMALGAM

To overcome the limitation of microleakage with amalgams, a coating of unfilled resin over the restoration margins and the adjacent enamel, after etching the enamel, has been tried. Although the resin may eventually wear away, it delays microleakage until corrosion products begin to fill the tooth restoration interface.

Mertz-fairhurst and others evaluated bonded and sealed composite restorations placed directly over frank cavitated lesions extending into dentin versus sealed conservative amalgam restorations and conventional unsealed amalgam restorations. The results indicate that both types of sealed restorations exhibited superior clinical performance and longevity compared with unsealed amalgam restorations over a period of 10 years.[45]

## FLUORIDATED AMALGAM

Fluoride, being cariostatic, has been included in amalgam to deal with the problem of recurrent caries associated with amalgam restorations. The problem with this method is that the fluoride is not delivered long enough to provide maximum benefit. Several studies investigated fluoride levels released from amalgam.[46–50] These studies concluded that a fluoride containing amalgam may release fluoride for several weeks after insertion of the material in mouth. As an increase of up to 10–20-fold in the fluoride content of whole saliva could be measured, the fluoride release from this amalgam seems to be considerable during the first week. An anticariogenic action of fluoride amalgam could be explained by its ability to deposit fluoride in the hard tissues around the fillings and to increase the fluoride content of plaque and saliva, subsequently affecting remineralization. In this way, fluoride from amalgam could have a favorable effect not only on caries around the filling but on any initial enamel demineralization. The fluoride amalgam thus serves as a “slow release device”.[47]

## BONDED AMALGAM

Conventional amalgam is an obturating material as it merely fills the space of prepared cavity, and thus, does not restore the fracture resistance of the tooth, which was lost during cavity preparations. In addition, the provision for adequate resistance and retention form for amalgams may require removal of healthy tooth structure. Further, since amalgam does not bond to tooth structure, microleakage immediately after insertion is inevitable. So, to overcome these disadvantages of amalgam, adhesive systems that reliably bond to enamel and dentin have been introduced.

Amalgam bond is based on a dentinal bonding system developed in Japan by Nakabayashi and co-workers.[51] The bond strengths recorded in studies have varied, approximately 12–15 MPa, and seem to be routinely achievable.[52–54] Using a spherical amalgam in one study of bonded amalgam, Summitt and colleagues reported mean bond strength of 27 MPa. The authors believed that this higher bond strength was achieved because the bonding material was refrigerated until immediately before its use.[55] Bond strengths achieved with admixed alloys tend to be slightly lower than those with spherical alloys.[56] One study compared post-insertion sensitivity of teeth with bonded amalgams to that of teeth with pin-retained amalgams. After 6 months, teeth with bonded amalgams were less sensitive than teeth with pin-retained amalgams. This difference in sensitivity was not present 1 year after insertion. This is possibly because of corrosion products in nonbonded amalgam restorations filling the interface, and thus, decreasing microleakage and sensitivity.[57]

If bonding proves successful over the long term, method of mechanical retention can be eliminated, thus reducing the potential for further damage to tooth structure that occurs with pin placement or use of amalgapins. If mechanical retention is not needed, cavity design can allow more sound tooth structure to be preserved.[41]

## CONSOLIDATED SILVER ALLOY SYSTEM

One amalgam substitute being tested is a consolidated silver alloy system developed at the National Institute of Standards and Technology.[58] It uses a fluoroboric acid solution to keep the surface of the silver alloy particles clean. The alloy, in a spherical form, is condensed into a prepared cavity in a manner similar to that for placing compacted gold. One problem associated with the insertion of this material is that the alloy strain hardens, so it is difficult to compact it adequately to eliminate internal voids and to achieve good adaptation to the cavity without using excessive force.[41]

## GALLIUM – AN ALTERNATIVE TO AMALGAM

Several times since the introduction of amalgam restorations to the United State in the 19th century, the public has expressed concerns about the use of mercury in dental amalgam. However, an effective alternative to amalgam has not been identified. As early as 1956, Smith and Caul[59–61] and Smith and co-workers[62] claimed that a gallium based alloy could serve as a possible alternative to dental amalgam. They found that mixing gallium with either nickel or copper and tin produced a pliable mass that could be condensed into a prepared cavity, which, after setting, had physical properties suitable for a restorative material.

## FUTURE OF DENTAL AMALGAM

The prediction that amalgam would not last until the end of the 20th century was wrong. Its unaesthetic appearance, its inability to bond tooth, concerns about the mercury and versatility of other materials have not not led to the elimination of this inexpensive and durable material. As other materials and techniques improve, the use of amalgam will likely continue to diminish, and it will eventually disappear from the scene.

Yet, amalgam continues to be the best bargain in the restorative armamentarium because of its durability and technique insensitivity. Amalgam will probably disappear eventually, but its disappearance will be brought about by a better and more esthetic material, rather than by concerns over health hazards. When it does disappear, it will have served dentistry and patients well for more than 200 years.

## CONCLUSIONS

Amalgam restorations have served the profession well and will continue to do so in the years to come. In terms of longevity, they are probably superior to composite resins, especially when used for large restorations and cusp capping. The new high copper single composition alloys offer superior properties but may not offer as good seal as older amalgams. The use of amalgam can be continued as a material of choice if esthetics is not a concern. Prepare a tooth as conservative as possible, making access large enough only for removal of carious dentin and using resin sealants for noncarious fissures.
